# Association between differences in ambient light exposure by hospital bed location and depressive symptoms among postpartum women

**DOI:** 10.3389/fpsyt.2026.1825979

**Published:** 2026-07-28

**Authors:** Hirofumi Hirakawa, Masaaki Muronaga, Mizuki Kudo, Shunsuke Yasumi, Eiji Kobayashi

**Affiliations:** 1 Department of Neuropsychiatry, Oita University Faculty of Medicine, Oita, Japan; 2 Department of Obstetrics and Gynecology, Oita University Faculty of Medicine, Oita, Japan

**Keywords:** ambient light, bed location, perinatal psychiatry, postpartum depression, the Edinburgh Postnatal Depression Scale

## Abstract

**Introduction:**

Environmental light is associated with the presence of depressive symptoms in healthy individuals, major depression, and postpartum depression. We hypothesized that women for delivery assigned to window-side beds (window-side group: WG) may exhibit lower levels of depressive symptoms at discharge than those assigned to corridor-side beds (corridor-side group: CG).

**Methods:**

This retrospective study involved postpartum women who were hospitalized for delivery in the obstetric ward of Oita University Hospital. We analyzed data of the Japanese version of the Edinburgh Postnatal Depression Scale (EPDS) assessed within approximately 1 week after delivery (within 7-days EPDS) and at an outpatient visit approximately 1 month after delivery (1-month EPDS). Analysis of covariance and binomial logistic regression analyses were conducted to evaluate between-group differences in EPDS scores and in the proportion of participants with EPDS scores of 9 or higher adjusting for relevant covariates. Pearson’s correlation analysis was used to assess the relationship between within 7-days and 1-month EPDS scores.

**Results:**

A significant between-group difference was observed for within 7-day EPDS scores, with mean scores of 3.4 and 5.0 in the WG and CG, respectively. In the binomial logistic regression analysis of within 7-days EPDS, bed location and cesarean delivery were significant in the final model. No significant between-group difference was observed in 1-month EPDS. A significant positive correlation was observed between the within 7-days and 1-month EPDS scores.

**Discussion:**

These findings suggest that increasing exposure to ambient light during hospitalization for delivery may help reduce depressive symptoms.

## 1 Introduction

Exposure to light, which enters the brain through the retina and is transmitted to various brain regions, exerts mood-enhancing effects (1). A significant negative correlation between the amount of daily exposure to environmental light and depressive scores has been previously reported in healthy participants, suggesting that ambient light may ameliorate low mood (2). A recent cross-sectional study revealed that greater exposure to bright light—defined as the daily amount of time to which an individual is exposed to more than 1,000 lux—was associated with fewer depressive symptoms (3). An association between environmental light and psychiatric symptoms has also been demonstrated, with depressive symptoms occurring more frequently during periods of a reduced number of hours of daylight (4). Furthermore, studies examining the association between postpartum depression and the duration of daylight have demonstrated that postpartum depression is more prevalent during periods of less exposure to daylight, both at 1 week postpartum and 4 to 6 weeks postpartum (5, 6). Together, these findings indicate that environmental light is associated with the presence of depressive symptoms in healthy individuals, major depression, and postpartum depression.

The effects of environmental light on psychiatric symptoms in relation to room location were previously examined among patients hospitalized for depressive episodes of bipolar disorder or for major depressive disorder: The length of hospital stay was compared between those admitted to rooms with windows facing east or southeast (allowing greater exposure to sunlight) and those admitted to rooms with windows facing west or northwest (allowing less exposure to sunlight). Patients hospitalized in rooms with east- or southeast-facing windows had significantly shorter lengths of stay (7, 8). These findings suggest that differences in exposure to environmental light based on the location of the hospital room may be associated with the rate of improvement in depressive symptoms, thereby potentially shortening the duration of hospitalization. To our knowledge, no studies have investigated the association between room or bed location and postpartum depression.

Based on the findings of these previous studies, we hypothesized that, among postpartum women hospitalized for delivery, exposure to environmental light differs between window-side and corridor-side beds, and that women assigned to window-side beds may exhibit lower levels of depressive symptoms at discharge than those assigned to corridor-side beds. The aim of the present study was to test this hypothesis using a retrospective study design.

## 2 Methods

### 2.1 Participants

This retrospective study involved postpartum women who were hospitalized for delivery in the obstetric ward of Oita University Hospital. The inclusion criteria were admission to a shared room in the obstetric ward (either a four- or two-bed room) and no change in bed location (window- or corridor-side) between admission and discharge. Participants were required to have completed assessments for postpartum depression at discharge and again at an outpatient visit 1 month postpartum. Because current or prior psychiatric disorders may strongly influence postpartum depressive symptoms (9), only women without a current presentation or history of psychiatric disorders were included. The exclusion criteria were admission to a single-bed room in the obstetric ward; any change in room (e.g., transfer from a shared room to a single-bed room) or bed location (window- or corridor-side) during hospitalization; and the presence or history of psychiatric disorders. Because cutoff scores of the Edinburgh Postnatal Depression Scale (EPDS) vary across countries (10), only Japanese participants were included. A previous study reported that the coronavirus disease 2019 pandemic affected depression and anxiety among pregnant and postpartum women (11), suggesting that the severity of depressive symptoms may differ from before and after the spread of this disease in Japan. Therefore, the study period was set to the 5 years from January 2015 to December 2019, prior to the outbreak of the coronavirus pandemic in Japan.

### 2.2 Setting

**Figure 1:**
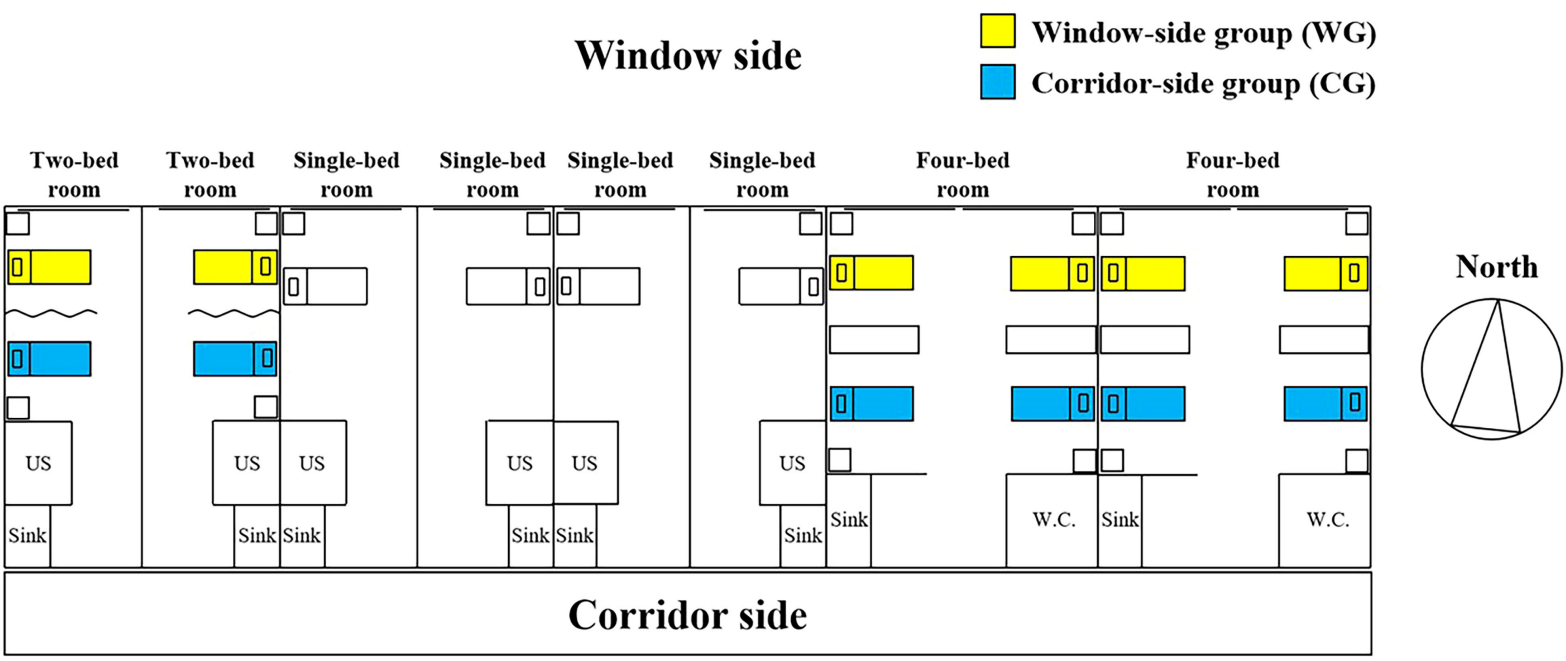
Bed rooms and bed location of this study. The obstetric ward of our hospital comprises two four-bed rooms, two two-bed rooms, and four single-bed rooms. Women assigned to the window-side beds formed the window-side group (WG; yellow beds) and those assigned to corridor-side beds formed the corridor-side group (CG; blue beds).

The obstetric ward of our hospital is located at 33°12′39″ north latitude and 131°32′4″ east longitude and comprises two four-bed rooms, two two-bed rooms, and four single-bed rooms. Women assigned to the window-side beds formed the window-side group (WG; yellow beds) and those assigned to corridor-side beds formed the corridor-side group (CG; blue beds) (**Figure 1**). As reference values, the illuminance levels at each bed, measured at the eye level of a seated patients using a lux meter at solar noon on a sunny day in May 2026, are shown in **Supplementary Figure 1** of the **Supplementary Material**.

### 2.3 Assessment of postpartum depression

The reliability and validity of the Japanese version of the EPDS have been verified (12). This version of the tool was used to screen for postpartum depression before discharge—that is, within approximately 1 week after delivery (within 7-days EPDS)—and at an outpatient visit approximately 1 month after delivery (1-month EPDS). The EPDS is a self-reported questionnaire developed to screen for postpartum depression (13). It consists of 10 items, each scored from 0 to 3, yielding a total score ranging from 0 to 30. The EPDS is widely used internationally and has been translated into 36 languages (10). Cutoff scores vary across countries; for example, the cutoff score of the original EPDS for probable postpartum depression was suggested to be 12 or 13 points, whereas that of the Japanese version, at 8 or 9 points, is lower (12, 13).

### 2.4 Data collection

We collected the EPDS scores for assessments conducted within 7 days postpartum and at 1 month postpartum, along with patient background data. Background data included age at delivery, body mass index (BMI) at admission, marital status, history of fertility treatment, history of miscarriage, twin pregnancy, obstetric complications, gynecological complications and medical comorbidities. Data on gestational age and delivery term (preterm, term, or post-term), mode of delivery (vaginal, vacuum-assisted, or cesarean section, including urgency), neonatal status (neonatal asphyxia, low birth weight infant, congenital anomalies, and admission to the neonatal intensive care unit), and serum hemoglobin levels at discharge, length of hospital admission were collected. Because this study focused on environmental light exposure, meteorological data for the city in which our hospital is located were obtained from the Japan Meteorological Agency (http://www.jma.go.jp/jma/index.html) for the periods during which each participant was hospitalized. Meteorological data included solar radiation, sunshine duration, temperature, precipitation, relative humidity, and atmospheric pressure. The authors assert that all procedures contributing to this work comply with the ethical standards of the relevant national and institutional committees on human experimentation and with the Helsinki Declaration of 1975, as revised in 2008. We uploaded our research proposal to our department’s website and patients were given the opportunity to decline consent for the use of their data. This opt-out study was approved by the Ethics Committee of the Oita University Faculty of Medicine on September 24, 2025 (approval number 3234).

### 2.5 Statistical analysis

The demographic characteristics of the WG and CG were compared using the two-sample t-tests and chi-square tests. The primary outcome was the group difference in within 7-days EPDS scores. The secondary outcomes were the group difference of the proportion of participants with within 7-days EPDS scores of 9 or higher, the group difference in 1-month EPDS scores and the proportion of participants with 1-month EPDS scores of 9 or higher. Both primary and secondary outcomes were analyzed using the two-sample t-tests and chi-square tests. With regard to between-group differences in EPDS scores, analysis of covariance was performed to adjust for factors previously reported in a meta-analysis to be associated with postpartum depression, including cesarean delivery, low birth weight infants, postpartum anemia (hemoglobin level), and obesity (BMI) (9). Because this study focused on environmental light exposure, solar radiation and sunshine duration during the hospitalization period were also included as covariates. Binomial logistic regression analysis using the likelihood ratio and forward selection method was performed to assess between-group differences in the proportion of participants with EPDS scores of 9 or higher, with an EPDS score of 9 or higher as the dependent variable and bed location (i.e., WG or CG) and the abovementioned covariates as independent variables. Pearson’s correlation analysis was used to assess the relationship between within 7-days and 1-month EPDS scores. The significance level was set at 0.05. Statistical analyses were performed using IBM SPSS version 28.0 (IBM Corp., Armonk, NY, USA).

## 3 Results

Among the 437 women who delivered at our hospital during the study period, 248 were admitted to and discharged from shared rooms. Of these, 99 patients who changed bed location between the window and corridor sides during hospitalization and 35 patients who were transferred between four- and two-bed rooms were excluded. Of the remaining 114 patients who were admitted to and delivered in shared rooms, and whose room and bed locations remained unchanged between admission and discharge, a further 14 patients with psychiatric disorders, 3 non-Japanese patients, and 11 patients with missing data for either the within 7-days or 1-month EPDS scores were excluded. Consequently, 86 patients (WG, n = 46; CG, n = 40) were included in the final analysis.

The mean age of the participants was 32.7 years, and the mean BMI at admission was 25.9 kg/m². Twenty-three women had a history of miscarriage, and twenty-four had a history of fertility treatment. Eighteen women delivered preterm, whereas sixty-eight delivered at term; no post-term deliveries were noted. Thirty-nine women underwent cesarean section. Twenty-four infants had a low birth weight. The mean hemoglobin level at discharge was 10.4 g/dL. The mean EPDS score within 7-days postpartum was 4.2, with 13 participants (15.1%) scoring 9 or higher. The mean EPDS score at 1 month postpartum was 3.7, with nine participants (10.5%) scoring 9 or higher. Detailed patient characteristics are presented in **Table 1**.

**Table 1:** The demographic characteristics of participants.

Demographic variables	All participants (n=86)
Age at delivery (s.d.)	32.7(6.0)
Body mass index at admission, kg/m2 (s.d.)	25.9 (5.1)
Gravidity (s.d.)	2.5 (1.5)
Parity (s.d.)	0.9 (0.9)
History of miscarriage (yes, no)	23, 63
History of fertility treatment (yes, no)	24, 62
Twin pregnancy (yes, no)	1, 85
Spouse (yes, no)	84, 2
Gestational age, days (s.d.)	263.6 (22.3)
Delivery term (preterm, term, post-term)	18, 68, 0
Mode of delivery (vaginal, vacuum-assisted, cesarean section)	43, 4, 39
Urgency of delivery (yes, no)	23, 63
Neonatal asphyxia (yes, no)	3, 83
Low birth weight infant (yes, no)	24, 62
Congenital anomalies of infant (yes, no)	2, 83
Admission to the neonatal intensive care unit (yes, no)	35, 51
Obstetric complications (yes, no)	61, 25
Gynecological complications (yes, no)	27, 59
Medical comorbidities (yes, no)	45, 41
Serum hemoglobin levels at discharge, g/dL (s.d.)	10.4 (1.4)
Length of hospital admission, days (s.d.)	10.4 (10.5)
Location of bed (window-side, corridor-side)	46, 40
Number of bed (four-bed, two-bed)	23, 63
Mean temperature, °C (s.d.)	16.4 (8.5)
Mean precipitation, mm (s.d.)	4.9 (6.6)
Mean sunshine duration, hr (s.d.)	5.5 (1.8)
Mean solar radiation, MJ/m2 (s.d.)	13.4 (4.4)
Mean relative humidity, % (s.d.)	71.2 (8.1)
Mean atmospheric pressure, hPa (s.d.)	1014.0 (6.3)
EPDS	
Within 7-days EPDS scores (s.d.)	4.2 (3.6)
EPDS scores of 9 or higher within 7-days (yes, no)	13, 73
One-month EPDS scores (s.d.)	3.7 (3.3)
EPDS scores of 9 or higher in one-month (yes, no)	9, 77

EPDS, the Edinburgh Postnatal Depression Scale; s.d., Standard deviation.

In the WG, the mean age of the participants was 33.4 years and the mean BMI at admission was 25.4 kg/m². Ten women had a history of miscarriage and thirteen had a history of fertility treatment. Ten women delivered preterm and twenty-two underwent cesarean section. Twelve infants had low birth weight. The mean hemoglobin level at discharge was 10.5 g/dL. In the CG, the mean age of the participants was 31.7 years and the mean BMI at admission was 26.5 kg/m². Thirteen women had a history of miscarriage and eleven had a history of fertility treatment. Eight women delivered preterm and seventeen women underwent cesarean section. Twelve infants had a low birth weight. The mean hemoglobin level at discharge was 10.4 g/dL. The demographic characteristics were similar between the WG and CG (**Table 2**).

**Table 2:** The demographic characteristics of window-side group and corridor-side group.

Demographic variables	Window-side group (WG; n= 46)	Corridor-side group (CG; n= 40)	P value
Age at delivery (s.d.)	33.4(6.0)	31.7(5.9)	0.17
Body mass index at admission, kg/m2 (s.d.)	25.4 (5.3)	26.5 (4.8)	0.31
Gravidity (s.d.)	2.7 (1.6)	2.3 (1.3)	0.21
Parity (s.d.)	0.9 (0.9)	0.8 (0.9)	0.5
History of miscarriage (yes, no)	10, 36	13, 27	0.26
History of fertility treatment (yes, no)	13, 33	11, 29	0.94
Twin pregnancy (yes, no)	1, 45	0, 40	0.54
Spouse (yes, no)	46, 0	38, 2	0.21
Gestational age, days (s.d.)	263.4 (23.6)	263.9 (21.0)	0.92
Delivery term (preterm, term)	10, 36	8, 32	0.84
Mode of delivery (vaginal, vacuum-assisted, cesarean section)	21, 3, 22	22, 1, 17	0.58
Urgency of delivery (yes, no)	10, 36	13, 27	0.26
Neonatal asphyxia (yes, no)	3, 43	0, 40	0.15
Low birth weight infant (yes, no)	12, 34	12, 28	0.69
Congenital anomalies of infant (yes, no)	1, 45	1, 39	0.72
Admission to the neonatal intensive care unit (yes, no)	20, 26	15, 25	0.57
Obstetric complications (yes, no)	31, 15	28, 12	0.8
Gynecological complications (yes, no)	13, 33	14, 26	0.5
Medical comorbidities (yes, no)	18, 28	13, 27	0.52
Serum hemoglobin levels at discharge, g/dL (s.d.)	10.5 (1.5)	10.4 (1.4)	0.89
Length of hospital admission, days (s.d.)	12.0 (13.8)	8.4 (3.3)	0.09
Type of room (four-bed, two-bed)	11, 35	12, 28	0.53
Mean temperature, °C (s.d.)	17.3 (9.4)	15.7 (7.5)	0.4
Mean precipitation, mm (s.d.)	5.1 (5.5)	4.5 (7.6)	0.7
Mean sunshine duration, hr (s.d.)	5.5 (1.8)	5.5 (1.8)	0.98
Mean solar radiation, MJ/m^2^ (s.d.)	13.5 (4.4)	13.3 (4.5)	0.79
Mean relative humidity, % (s.d.)	71.2 (8.1)	71.4 (8.2)	0.9
Mean atmospheric pressure, hPa (s.d.)	1013.0 (6.9)	1014.8 (5.6)	0.19

CG, Corridor-side group; EPDS, the Edinburgh Postnatal Depression Scale; s.d., Standard deviation; WG, Window-side group.

**Figure 2:**
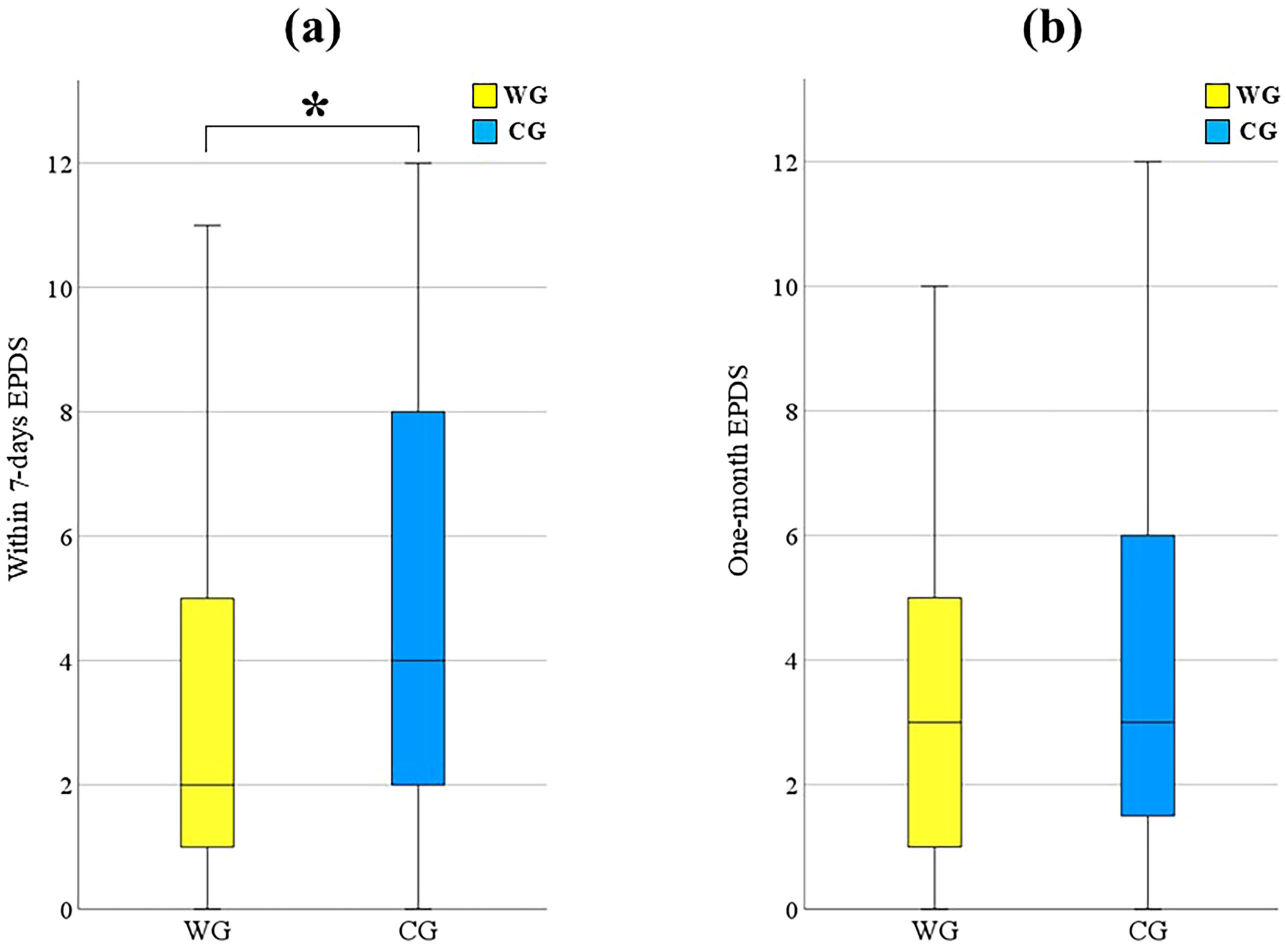
Difference of the EPDS between window-side group and corridor-side group. **(a)** A significant between-group difference was observed for within 7-day EPDS scores in the WG (represented by yellow) and CG (represented by blue). **(b)** There was no significant between-group difference in the 1-month EPDS scores in the WG and 4.2 in the CG (p = 0.22). *Indicates that the p values < 0.05. Unit shower, US; Water Closet, W.C.

A significant between-group difference was observed for within-7-day EPDS scores, with mean scores of 3.4 and 5.0 in the WG and CG, respectively (p = 0.045). The between-group difference in the EPDS scores remained significant after adjusting for the covariates (p = 0.042; **Figure 2a**; **Table 3**). A trend toward a higher proportion of participants with EPDS scores of 9 or higher was noted in the CG compared with the WG (4 [8.7%] vs. 9 [22.5%], respectively; p = 0.08). Binomial logistic regression analysis showed that bed location (Odds ratio:0.25; 95% CI: 0.06-0.98; p=0.047) and cesarean section (Odds ratio:0.1; 95% CI:0.02-0.49; p=0.005) were significant in the final model.

**Table 3:** Difference of the EPDS between window-side group and corridor-side group.

	Window-side group (WG; n= 46)	Corridor-side group (CG; n= 40)	P value
Within 7-days EPDS scores (s.d.)	3.4 (3.0)	5.0 (4.0)	0.042*
One-month EPDS scores (s.d.)	3.3 (3.0)	4.2 (3.7)	0.24

CG, Corridor-side group; EPDS, the Edinburgh Postnatal Depression Scale; s.d., Standard deviation; WG, Window-side group.

Covariates: cesarean delivery, low birth weight infants, serum hemoglobin levels at discharge, body mass index at admission, solar radiation and sunshine duration during the hospitalization period.

*Indicates that the p values < 0.05

We observed no significant between-group difference in the 1-month EPDS scores, with a mean score of 3.3 in the WG and 4.2 in the CG (p = 0.22). This finding remained nonsignificant after adjusting for covariates (p = 0.24; **Figure 2b**; **Table 3**). Similarly, no significant difference in the proportion of participants with EPDS scores of 9 or higher was observed between the WG and CG (3 [6.5%] vs. 6 [15.0%], respectively; p = 0.18). Binomial logistic regression analysis revealed that none of the covariates, including bed location, showed a statistical significance.

**Figure 3:**
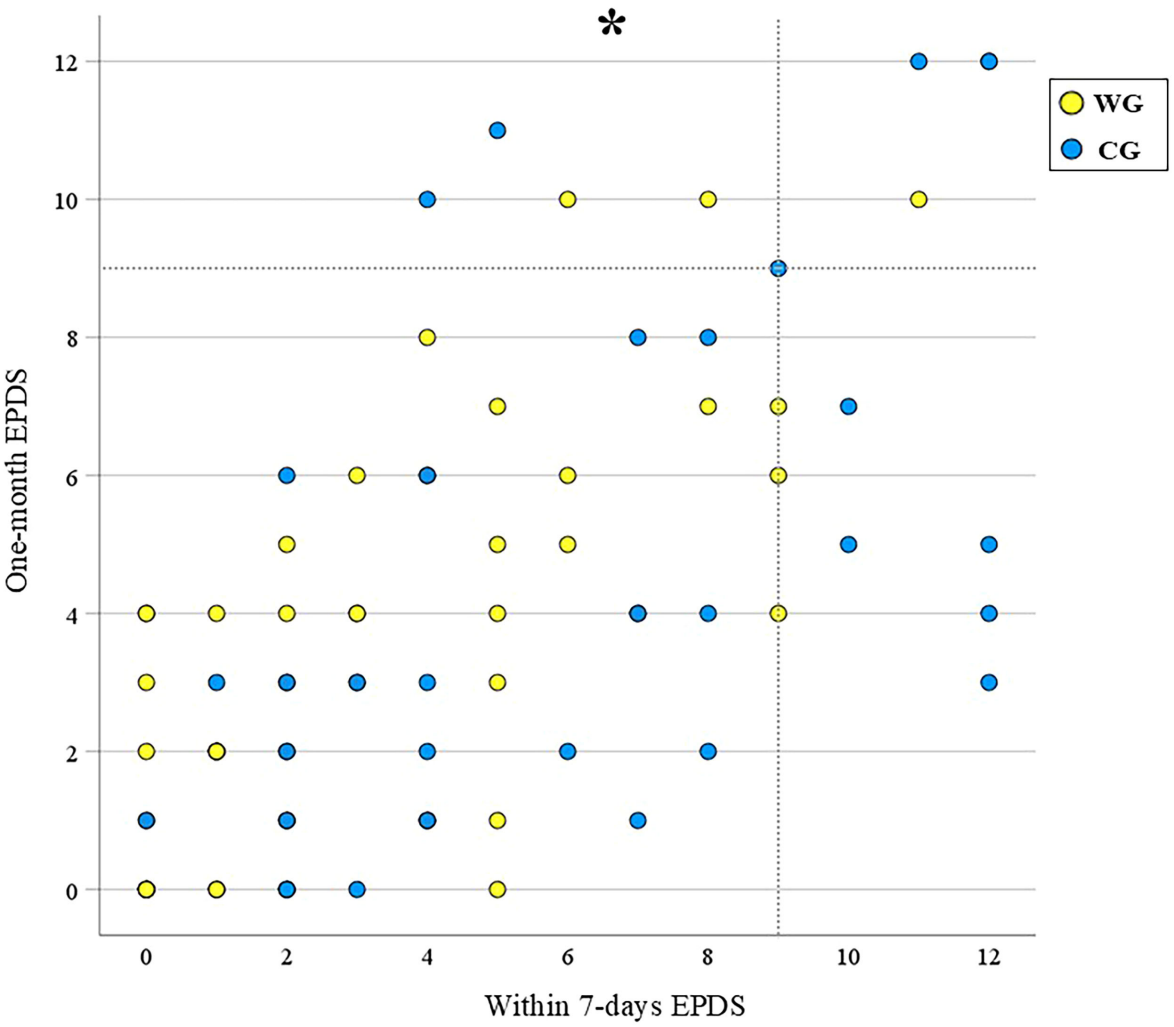
Association between within 7-days and 1-month EPDS scores. Yellow circle represents the WG while blue circle represents the CG. The gray dashed line indicates the cutoff score of 9 on the Japanese version of the EPDS.A significant positive correlation was observed between the within 7-days and 1-month EPDS scores (r = 0.65; p < 0.001) in all participants. In subgroup analyses, significant positive correlations between the within 7-days and 1-month EPDS scores were observed in both the WG and CG (r = 0.66; p < 0.001 and r = 0.64; p < 0.001, respectively). *Indicates that the p values < 0.05.

Across all participants, a significant positive correlation was observed between the within 7-days and 1-month EPDS scores (r = 0.65; p < 0.001; **Figure 3**). In subgroup analyses, significant positive correlations between the within 7-days and 1-month EPDS scores were observed in both the WG and CG (r = 0.66; p < 0.001 and r = 0.64; p < 0.001, respectively).

## 4 Discussion

In the present study, our findings demonstrated a significant difference in the EPDS scores within 7 days of delivery between women assigned window- or corridor-side beds. Also, the proportion of participants with EPDS scores of 9 or higher within 7-days postpartum was significantly associated with bed location after adjusting for covariates. The present results are consistent with our hypothesis. To the best of our knowledge, this is the first study to investigate the association between differences in exposure to ambient light according to hospital bed location and depressive symptoms among postpartum women.

An estimated 10% to 20% of women experience depression during the perinatal period (14, 15). Both prenatal and postpartum depression have been linked to a variety of adverse outcomes, including impaired mother–infant bonding; they have also been shown to affect childhood social and cognitive development and be associated with behavioral problems such as child abuse and suicide (16, 17). Therefore, preventing depression during pregnancy and the early postpartum period, as well as implementing early interventions, is beneficial for both mothers and children; such prevention is critically important. The EPDS is a useful and recommended screening tool for use during outpatient visits at 4–8 weeks postpartum (15, 17). Several studies have reported significant associations between EPDS scores assessed in the early postpartum period (e.g., day 2, 1 week, or 2 weeks postpartum) and those assessed at 4 weeks postpartum (15, 17, 18). Consistent with these findings, our results also demonstrated a positive correlation between EPDS scores within 7 days postpartum and those at 1 month postpartum.

Because most psychotropic medications cross the placenta during pregnancy and, postpartum, are excreted into breast milk, the use of non-pharmacological interventions is generally preferred during the perinatal period. Lifestyle interventions such as physical activity and dietary or nutritional supplementation are known to reduce the risk of perinatal depression; however, the extent to which ambient light exerts a preventive effect on perinatal depression remains unclear (19–21). A variety of non-pharmacological treatments for perinatal depression have also been reported: These interventions include psychological interventions such as cognitive behavioral therapy, as well as complementary and alternative approaches such as physical activity, yoga, intake of fatty acids, Chinese herbal medicine, music therapy, and bright light therapy (19). Among these approaches, bright light therapy is an effective, well-accepted, safe, and low-cost non-pharmacological treatment with a highly favorable risk to benefit ratio for depressive disorders (22). Recently, we recommended light modulation therapy using bright light therapy as an adjunctive non-pharmacological treatment for perinatal bipolar disorder in the clinical practice guideline for managing perinatal mood, anxiety, and related disorders by the Canadian Network for Mood and Anxiety Treatments (23, 24). Although light therapy has been shown to alleviate depressive symptoms, it typically requires patients to sit in front of a light device for 30 minutes to several hours (25). Therefore, as a simpler addition to daily routines, we have previously recommended that patients with depressive symptoms increase their exposure to ambient light, for example, by opening curtains in the morning or walking outdoors (26).

In the present study, we observed significant differences in EPDS scores and the proportion of participants with EPDS scores of 9 or higher between the WG and the CG within 7-days postpartum after adjusting for covariates; however, this difference was no longer statistically significant at 1-month postpartum. Because the within 7-days EPDS scores were assessed immediately before discharge, the observed difference may be attributable to differences in exposure to ambient light during hospitalization. By contrast, at 1-month postpartum participants were attending outpatient follow-up visits; therefore, the differences in exposure to ambient light during daily life may have diminished between the two groups. This may potentially explain the absence of a significant between-group difference at 1 month. Nevertheless, our findings suggest that, even in the absence of a significant difference at 1 month postpartum, assignment to a window-side bed may be associated with lower depressive symptoms among postpartum women, particularly in the immediate postpartum period. Our findings suggest that allocating pregnant women at high risk for postpartum depression to window-side beds during hospitalization for delivery may increase their exposure to ambient light and help reduce depressive symptoms. Because this approach simply involves paying attention to bed location for perinatal women, it is very practical, cost-free, and carries no risk of adverse effects; therefore, we consider it to be a clinically valuable strategy.

Our study had several limitations. First, because this was a retrospective rather than an interventional study, there may have been potential selection bias. Second, this study inferred greater light exposure among women assigned to window-side beds based solely on bed location; the actual amount of light exposure experienced by each woman during hospitalization was not directly measured. Third, although EPDS scores were assessed at discharge and during outpatient follow-up, EPDS scores before delivery were not evaluated; baseline depressive symptoms for each woman could not be determined. Therefore, it is difficult to determine whether the observed differences are due to the exposure or to pre-existing differences between the groups. Fourth, other environmental differences between bed locations, such as window view content and noise levels, were not controlled for and may have independently influenced mood. Finally, the relatively small sample size may limit statistical power, particularly for multivariable analyses, and may also limit the generalizability of the findings.

In conclusion, in the present study, we demonstrated a significant difference in the total scores for the EPDS assessed within 7-days of delivery between women assigned to window- or corridor-side beds. Also, the proportion of probable postpartum depression within 7-days was significantly associated with bed location after adjustment for covariates. These findings suggest that increasing exposure to ambient light during hospitalization for delivery may help reduce depressive symptoms. Further studies are warranted to clarify the association between ambient light exposure and postpartum depression.

## Data Availability

The data analyzed in this study is subject to the following licenses/restrictions: the datasets analyzed during the current study are available from the corresponding author on reasonable request.
