# Supplementary material for: Association between differences in ambient light exposure by hospital bed location and depressive symptoms among postpartum women
Source: Front Psychiatry. 2026 Jul 28;17:1825979. doi: 10.3389/fpsyt.2026.1825979 (PMC13457735; doi:10.3389/fpsyt.2026.1825979)
Supplement: Supplementary file 1 [file SupplementaryFile1.docx]

Supplementary Material

# 1 Supplementary Figure

# Figure S1 Differences in ambient light exposure between window-side and corridor-side beds.

The illuminance levels at each bed were measured at the eye level of a seated patient using a lux meter at solar noon on a sunny day in May 2026. Yellow beds represent the window-side group, whereas blue beds represent the corridor-side group.
